# Time to first antenatal care booking and its determinants among pregnant women in Ethiopia: survival analysis of recent evidence from EDHS 2019

**DOI:** 10.1186/s12884-022-05270-1

**Published:** 2022-12-09

**Authors:** Tegene Atamenta kitaw, Ribka Nigatu Haile

**Affiliations:** School of Nursing, College of Health Science, Woldia University, Woldia, Ethiopia

**Keywords:** Antenatal care, Determinants, Ethiopia

## Abstract

**Background:**

Antenatal care is one of the components of the maternal and child continuum of care. Timely booking ANC during pregnancy is of utmost importance to guarantee the best possible health outcomes for women and children. Inappropriate timing of the first ANC booking is associated with poor pregnancy outcomes, including perinatal death, stillbirth, and early neonatal death. According to WHO focused ANC recommendation, every mother should start booking ANC within the first 12 weeks of gestational age. However, in developing countries, including Ethiopia, many pregnant mothers were not booking ANC at the recommended time. Thus, this study aims to assess the time to first ANC booking and its determinants in Ethiopia.

**Methods:**

A survival analysis was conducted to determine time to first ANC booking among 3917 weighted study subjects. The data were extracted from EDHS 2019 using STATA version 17 software. A Kaplan Meier survivor curve was computed to estimate the time of first ANC booking. A Long-rank test was used to compare the difference in survival curves. Weibull Inverse Gaussian shared frailty model was used to identify significant predictors. On multivariable analysis, variables having a *p*-value of ≤ 0.05 are considered statistically significant.

**Results:**

The overall median survival time was four months. The significant determinant of time to first ANC booking are residence (rural [ϕ = 1.111, 95CI: 1.060, 1.164), mother educational level (primary education [ϕ = 0.945, 95CI: 0.915, 0.977], secondary and above educational [ϕ = 0.857, 95CI: 0.819, 0.896]) and wealth index (middle [0.948 (ϕ = 0.948, 95CI: 0.911, 0.986) and rich [ϕ = 0.904, 95CI: 0.865, 0.945])

**Conclusion:**

The median time for first ANC booking is 4 month, which is higher than the WHO recommended time. The timing of the first ANC booking in Ethiopia was mainly influenced by the residence of women, mother educational level, and wealth index. It is strongly recommended to expose mothers to educational materials and other awareness-creation campaigns, as well as to support disadvantaged women, such as the uneducated, poor, and those living in rural or remote areas.

## Background

Antenatal care (ANC) is one of the components of the maternal and child continuum of care [1]. ANC is the care provided by health care professionals to pregnant women to ensure the optimal health condition for both mother and fetus throughout pregnancy. The elements of ANC consist of risk detection, prevention, and treatment of pregnancy-related disorders, as well as health education and health promotion [2]. According to WHO Focus antenatal care model recommendation, all pregnant mothers are better to start ANC booking within the first 12 weeks of gestational age [3]. Globally, the prevalence of early ANC bookings was 43%. There is a significant disparity across regions. The prevalence of early ANC booking was 85%, below 45% and less than 25% in developed, developing, and sub-Saharan regions respectively [4]. In Ethiopia, Only 20% of pregnant mother had their first ANC during the first trimester of pregnancy [5].

Every day approximately 830 women die from preventable causes related to pregnancy and childbirth. 99% of all maternal deaths occur in developing countries. Among developing countries Sub-Saharan Africa is the leading in count with 546 maternal death per 100,000 live births [6]. In Ethiopia the mortality rate was 412 maternal deaths per 100,000 live births [7].

The time at first ANC visit determines the women’s and children’s health later in life. Timely first ANC booking improves the possibility of adherence to the clinically indicated check-up schedule while giving healthcare practitioners sufficient time to recognize and successfully treat diseases such as syphilis, anemia, malaria, and hypertension [8, 9]. Booking the first ANC appointment within the first three months of pregnancy is especially important in the treatment of health issues that may lead to complications for mother and baby later on [10, 11]. One of the main reasons for higher maternal mortality due to pregnancy complications in Sub-Saharan Africa than in any other part of the world is the inaccessibility and delayed initiation of ANC visits [12].

Delayed initiation of first ANC visit is associated with poor pregnancy outcomes, including perinatal death, stillbirth, and early neonatal death [13]. It is also linked with an increase in the risk of dying during pregnancy and childbirth, as well as long and short term maternal complications [14]. About 25% of maternal deaths occur during the ANC period, primarily owing to pre-eclampsia, eclampsia, and antepartum hemorrhage, which can be either prevented or treated during ANC period [15]. On the other hand, about 9.2 fetal death per 1000 births occur in Ethiopia during the ANC period [16].

According to several studies, the determinants of time to first ANC booking were maternal education [17–19], maternal age [20], parity [21, 22], occupational status [23, 24], wealth status [17, 25], husband education [18, 26], and distance from health facility [26, 27], marital status [28], residence [29] and religion [28]. Furthermore, health care provider and hospital related factors such as gender insensitivity in providing care through male health workers, and cost/time in ANC visits are also reported as barrier for early initiation of ANC [30].

By 2030, one of the target of Ending Preventable Maternal Mortality (EPMM) is to lower the global maternal mortality ratio (MMR) to under 70 per 100,000 live births. Countries should lower their MMRs from their 2010 baseline by at least two-thirds. No country should have an MMR above 140 maternal deaths per 100,000 live births by the year 2030 [31]. Even though Ethiopia has reduced maternal and child mortality by half through implementing a free service package for maternal health care service, maternal mortality rate of 412 per 100,000 live births and child mortality rate of 67 per 1,000 are still too high. Promising progress has also been made, with half of the mothers giving birth in a health institution [32]. However, the timely initiation of maternal health services remains a challenge.

In achieving the above goals, the timely booking of ANC plays a paramount role. A few related studies have been undertaken so far in Ethiopia. Most studies focus on how many pregnant mothers have delayed their first ANC visit and the number of ANC visits throughout pregnancy. The majority of the studies are limited to certain districts. Survival analysis of time to first ANC booking will have an enormous role in overcoming those limitations and further estimating the substantial impact of predictor variables at a national level.

Understanding the time to first ANC booking has a crucial role in providing information for program planners and policymakers so as to prevent the consequence of inappropriate timing of first ANC booking. In addition, it is also essential to take into account the time at first booking in addition to the number of ANC visits during pregnancy. Furthermore, this study uses recent (2019) Ethiopia Demographic and Health Survey (EDHS) data, which has a paramount role in giving current information to know improvement regarding time at first ANC visit.

## Methods

### Study setting, study period and data source

According to the latest census figures and projections from trading economics, the Ethiopian population was estimated at 115.0 million in 2020 [33]. The EDHS report presents comprehensive, detailed, final outcomes of the survey at the national level, for the nine regional states and two city administrations of Ethiopia. The administration levels went from regions to zones and through woreda. A survival analysis study was conducted among pregnant women in Ethiopia using the EDHS data. The EDHS was implemented by the Ethiopian Public Health Institute (EPHI) in partnership with the Central Statistical Agency (CSA) and the Federal Ministry of Health (FMoH). The target groups were women age 15–49 and men age 15–59 in randomly selected households across Ethiopia. EDHS contains information on the background characteristics of the respondents, maternal health care, fertility, marriage and sexual activity, child feeding practices, nutritional status of women and children, and adult and childhood mortality. Data collection lasted from March to June 2019 [34].

### Data extraction, population

After a reasonable request, we receive a permission letter to download EDHS 2019 data from DHS program. Data extraction was done to select pregnant mothers. 3,979 pregnant mothers were extracted, from whom 17 were unknown dates of ANC visit. The final extracted sample size was 3962. After weighting, the final extracted weighted sample size was 3,916.7 (3917). The data extraction period was from May 1 to June 1, 2022. All pregnant women five years preceding the survey were the source population, whereas all pregnant women five years preceding the survey in the selected enumeration area were the study population.

### Sampling methods

**Fig. 1:**
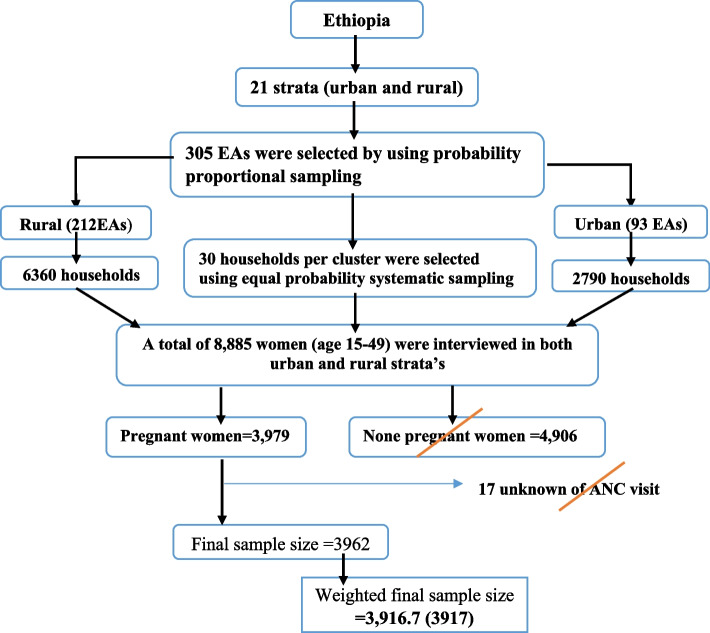
Schematic representation of the sampling procedures in the study of time to first ANC Booking and its determinants among pregnant women in Ethiopia, EDHS, 2019. N.B EAs = Enumeration Areas

The 2019 EDHS sample was stratified and selected in two stages. Each region was stratified into urban and rural areas, yielding 21 sampling strata. In the first stage, a total of 305 EAs (93 in urban areas and 212 in rural areas) were selected with probability proportional to EA size. In the second stage, a fixed number of 30 households per cluster were selected with an equal probability systematic selection from the newly created household listing. The detailed sampling procedure is available in the EDHS 2019 report [34]. In this study, a total of 3917 weighted pregnant mother were included. The highlighted sampling procedure for this study is indicated in the figure below (Fig. 1).

### Inclusion and exclusion criteria

All pregnant women with the age group of 15–49 and whose gestational age was known at first ANC booking were included. Furthermore, women who did not receive ANC throughout pregnancy and gestational age recorded at delivery or termination of pregnancy were also included in the study. However, women whose gestational age at the time of first ANC booking is unrecorded or unknown were excluded from the study

### Study variables

The dependent variable is the time (in month) at first ANC booking. This study considered different explanatory variables to determine predictors of time to first ANC booking (Table 1).

**Table 1:** List of explanatory variables for the assessment of time to first ANC booking in Ethiopia

Variable	Descriptions
Woman’s age	The ages of pregnant mother was categorized as 15–24,25–34, 35–49
Residence	Women’s residence place was Urban or Rural
Mother Educational level	Women level of education was classified as: No education, Primary, Secondary or higher
Wealth index	It was categorized as Poor, Middle, and Rich
Marital status	Not married or Married
Parity	The number of children ever born including the current pregnancy categorized as: 2 or less and 3 and above
Sex of household head	Head of household categorized as male or female
Household size	Household size classified as: 1–4, 5–9 and 10 and more

### Operational definition

#### Event

If pregnant had at least one ANC visit during pregnancy.

#### Censored

If pregnant mother did not receive ANC throughout pregnancy and gestational age or duration of pregnancy is recorded during delivery or termination of pregnancy.

#### Survival time

The time it takes to have first ANC booking in month from the date of pregnancy to first ANC booking (gestational age at first ANC booking).

### Data processing and analysis

STATA version 17 software was used to extract data from the EDHS 2019 individual (women) record folder. The data was coded, cleaned, and edited. Listing and sorting were done to identify any missing values. Descriptive statistics are analyzed and presented in terms of frequency and percentage. A Kaplan Meier survivor curve was computed to estimate the time of first ANC booking. A long-rank test was used to compare the difference in survival curves between categories of variables. Akaike information criteria (AIC) and Bayesian information criteria (BIC) was computed to select appropriate survival model for the data. Multicollinearity was checked prior to running selected survival model. The variance inflation factor result showed that the maximum VIF was 1.61 for wealth index and the mean VIF was 1.31. Based on the VIF result, there is no multicollinearity between covariates. Proportional hazard assumption test was checked by using Shenfield residuals. Variables having a *p*-value of ≤ 0.25 in the bivariate analysis were fitted and included in the multivariable Weibull Inverse Gaussian shared frailty model.In multivariable analysis; those variables having a *p*-value of ≤ 0.05 are considered statically significant.

### Rationale for using the survival analysis

Since the event of interest in this study is time to first ANC booking, an outcome variable of interest time until an event occurs; it is best suited to using a survival analysis model. Besides, survival analysis is a statistical procedure that is significant to determine the relationship between an explanatory variable and survival time (time to first ANC booking).

## Results

### Socio-demographic and obstetric characteristics

3917 weighted study subject who were pregnant during five years survey were included to examine the time to first ANC booking. 2913 (74.4%) of pregnant women’s had at least one ANC visit. Weighted frequency analysis showed that 2897 (74.0%) of respondents resided in rural areas. Regarding educational status, 2010 (51.3%) of respondents have no formal education. 1646 (42.0%) of the pregnant mother were in the poor household wealth index category (Table 2).

**Table 2:** Socio-demographic and obstetric characteristics of pregnant women in Ethiopia, EDHS 2019

Variable	Categories	Weighted frequency	First ANC booking status	
			Censored (%)	Event (%)
**Age**	15–24	994	237(6.1%)	757 (19.3%)
	25–34	1987	442 (11.3%)	1545(39.4%)
	35–49	936	324 (8.3%)	612(16.6%)
**Residence**	Urban	1020	155 (3.9%)	865(22.1%)
	Rural	2897	848 (21.7%)	2048(52.3%)
**Mother Educational level**	No education	2010	732(18.7%)	1278(32.6%)
	Primary	1411	261 (6.7%)	1150 (29.4%
	Secondary and above	496	10 (0.3%)	486(12.4%)
**Wealth index**	Poor	1646	662(16.9%)	984(25.1%)
	Middle	761	173 (4.4%)	588 (15.0%)
	Rich	1510	170 (4.3%)	1340 (34.2%)
**Marital status**	Not married	269	81 (2.1%)	187(4.8%)
	Married	3648	922(23.5%)	2726 (69.6%)
**Parity**	2 or less	3590	854 (21.8%)	2736 (69.8%)
	3 and above	327	150(3.8%)	177 (4.5%)
**Sex of household head**	Male	3394	863 (20.0%)	2531 (64.6%)
	Female	523	140 (3.6%)	382 (9.8%)
**Household size**	1–4	1299	229 (5.8%)	1070 (27.3%)
	5–9	2381	670 (17.1%)	1710 (43.7%)
	10 and more	237	104 (2.7%)	133(3.4%)

### Survival time of first ANC booking

**Fig. 2:**
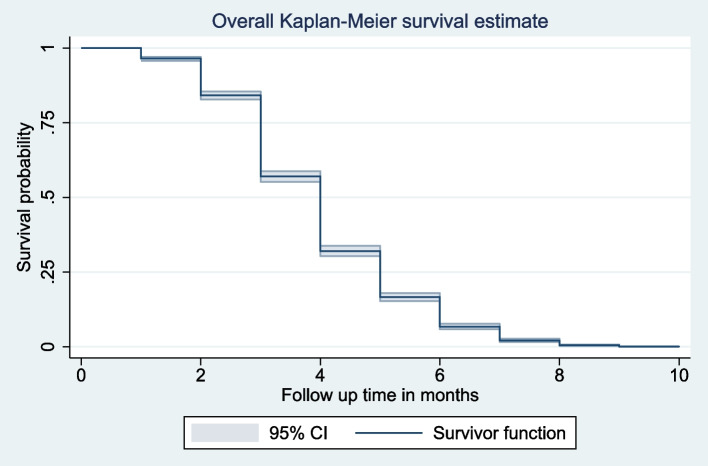
Overall Kaplan–Meier failure curve of time to first ANC booking in Ethiopia, EDHS 2019

The overall median survival time to first ANC booking was four month. The total follow-up time contributed by all study participants was 11,441-person months. The survival probability of time to first ANC booking among pregnant women’s beyond 2, 4, 6 and 8 month was 96.37%, 56.98%, 16.59% and 2.11% respectively (Fig. 2).

The median survival time was quite varied among the participant characteristics. The median survival time was 4 month for no education and 3 month for those with secondary and above educational level. By wealth index, the median survival times for the poor, middle, and rich were 4, 4, and 3 months, respectively. The median survival time by age and parity does not show a significant difference.

### Comparisons of survival functions of different categorical variables

Kaplan–Meier survival curve and Log-rank test were computed to compare and estimate the survivor function among different groups of variables. In the Kaplan–Meier survival curve, one survivorship function curve located under another means the lower curve group has a lower survival status than the upper curve group or has a less desirable survival probability than the upper curve. Furthermore, the difference was described statistically by the log-rank test.

**Fig. 3:**
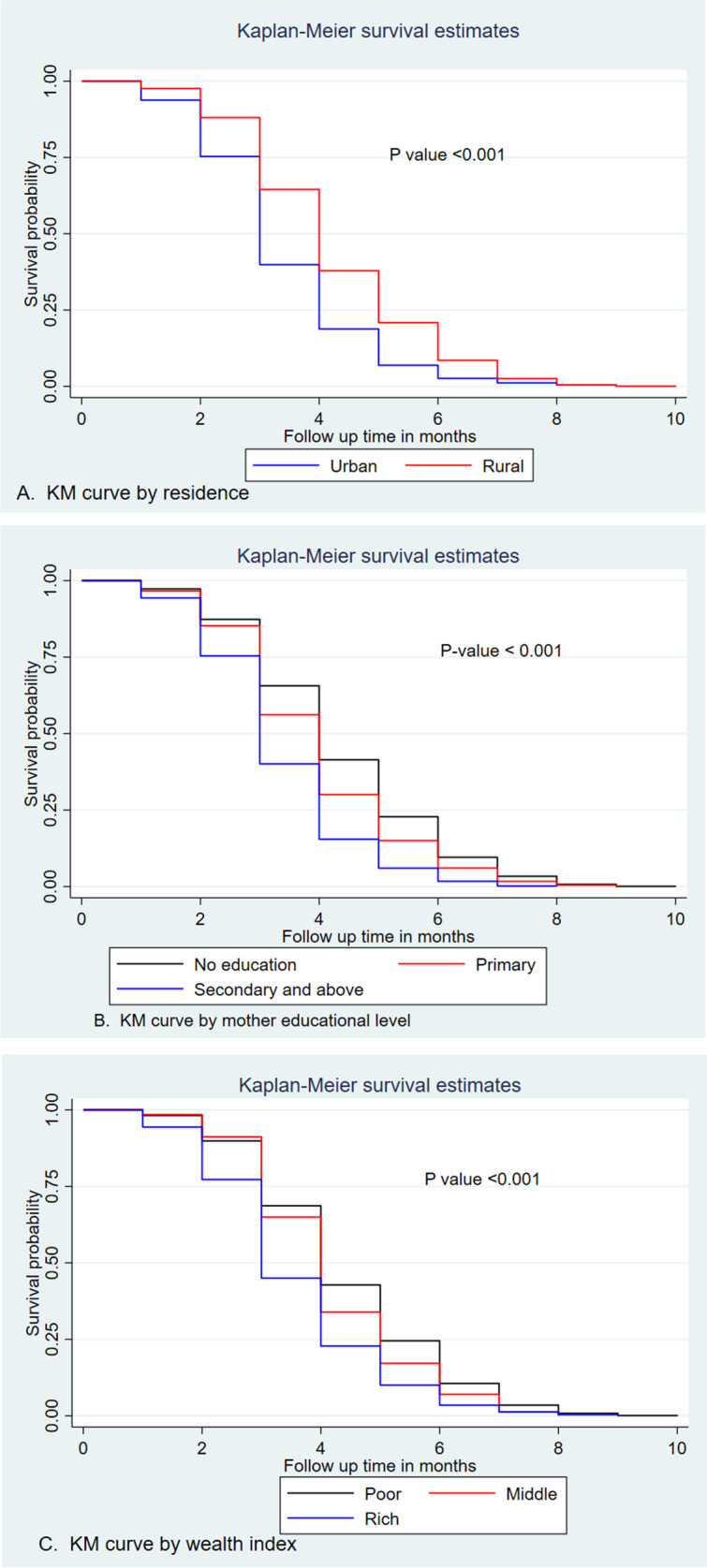
Kaplan–Meier survival curves and log rank tests of time to first ANC booking among pregnant women by their characteristics, EDHS 2019

Generally, Kaplan–Meier survival curve shows that pregnant mother who reside in rural area, no formal education and poor wealth index categories starts first ANC visit lately than the reverse group. Furthermore, the Log-rank test revealed that there is a statistically significant difference in survival experience among covariates of residence (*P* value < 0.001), mother educational level (*P* value < 0.001) and wealth index (*P* value < 0.001) (Fig. 3).

### Model selection

#### Proportional hazard assumption test by schoenfeld residual

The rho statistic p value of all covariates and global test in schoenfeld residual test is below 0.05. Which means that the proportional hazard assumption is not satisfied. Accelerated failure time model should be considered.

#### Accelerated failure time model test

The model with the smallest AIC and BIC values was considered to be the best survival model for the given data. Weibull Inverse Gaussian shared frailty model was found to have the smallest AIC and BIC values (2869.56 and 2935.23). Therefore, Weibull Inverse Gaussian shared frailty was the best model for the data to describe the status of pregnant mother toward time to first ANC booking and its determinants (Table 3).

**Table 3:** Comparison of akaike information criteria and bayesian information criteria among different accelerated failure time model and frailty distribution

Information criteria	Models	Frailty distributions		
		**No frailty**	**Gamma frailty**	**Inverse Gaussian frailty**
**AIC**	Exponential	6252.84	6254.84	6252.84
	Weibull	2915.84	2873.45	**2869.56**
	Log-logistic	3043.70	3045.70	3043.70
	Log-normal	3140.41	3142.41	3140.41
**BIC**	Exponential	6306.57	6314.54	6306.57
	Weibull	2975.53	2939.11	**2935.23**
	Log-logistic	3103.4	3111.37	3103.4
	Log-normal	3200.11	3208.08	3200.11

**NB: AIC = **akaike information criteria and **BIC = **bayesian information criteria

#### Determinants of time to first ANC booking

In the bivariable weibull inverse gaussian shared frailty woman’s age, residence, marital status**,** women’s education level, parity**,** wealth index and sex of household head were found to be significant at *p*-value ≤ 0.25. In the multivariable weibull inverse gaussian shared frailty model residence, mother educational level and wealth index were found to be determinants of time to first ANC booking.

The acceleration factor for time to first ANC booking among pregenant women who reside in rular area was 1.111 (ϕ = 1.111, 95CI: 1.060, 1.164) when compared to pregnant women who were from urban area. This shows that pregnant women from rural areas starts first ANC booking lately as compared to pregnant women from urban areas. Pregnant women who attend secondary and above educational levels accelerate the time to first ANC booking by factors of 0.857 (ϕ = 0.857, 95CI: 0.819, 0.896) as compared to the reference group (no education). Furthermore, women who attend primary educational level accelerate the time to first ANC booking by a factor of 0.945 (ϕ = 0.945,95CI: 0.915, 0.977) when compared to uneducated women. Pregnant women who were in household wealth index categories of middle and rich had 0.948 (ϕ = 0.948,95CI: 0.911,0.986) and 0.904 (ϕ = 0.904,95CI: 0.865,0.945) times shorter survival times compared to women in poor wealth index categories, respectively (Table 4).

**Table 4:** Bivariable and multivariable Weibull inverse gaussian shared frailty analysis for determinants of time to first antenatal booking in Ethiopia, EDHS 2019(n = 3917)

Variable	Categories	Coef	Acceleration factor *(ϕ)*	95% CI for *ϕ*	*P*-value
Age	15–24			1	1
	25–34	-0.0152	0.985	[0.956, 1.015]	0.317
	35–49	-0.0057	0.994	[0.951, 1.039]	0.801
Residence	Urban			1	1
	Rural	0.1051	1.111	[1.060, 1.164]	**0.000**
Mother Educational level	No education			1	1
	Primary	-0.0561	0.945	[0.915, 0.977]	**0.001**
	Secondary and above	-0.1546	0.857	[0.819, 0.896]	**0.000**
Wealth index	Poor			1	1
	Middle	-0.0536	0.948	[0.911, 0.986]	**0.008**
	Rich	-0.1004	0.904	[0.865, 0.945]	**0.000**
Marital status	Not married			1	1
	Married	-0.0036	0.996	[0.942, 1.054]	0.900
Parity	2 or less			1	1
	3 and above	0.0519	1.053	[0.993, 1.117]	0.083
Sex of household head	Male			1	1
	Female	-0.0345	0.966	[0.925,1.009]	0.117

## Discussion

Time to first ANC booking and its determinants was determined by using the recent 2019 Ethiopian Demographic Health Survey data. In this study, the median survival time was four months. Residents, mother's educational level, and wealth index were found to be a significant determinant of time to first ANC booking.

The overall median time to first ANC booking is four month. This finding is in line with the study done in Addis Ababa (4 month) [35]. However, the above finding is lower than the study done in Central Zone, Tigray (5 month) [36], Arba Minch town and district (5 month) [17], Tanzania (5 month) [37], Nigeria(6 month) [38], previous study from EDHS 2016 (7 month) [39] and Uganda (7 month) [40]. This variation could be attributed to the fact that recently, in Ethiopia, different interventions were done in line with the Ethiopian national reproductive health strategy with the primary outcome target of 95% of pregnant women starting the first antenatal care visit before 16 weeks’ gestation and having at least 4 antenatal care visits. The main interventions include: increasing the community’s awareness of the importance of birth preparedness; ensuring the provision of optimal clinical care in all health facilities during pregnancy; strengthening the primary level health care linkage and referral system; and expanding the prevention of mother-to-child transmission (PMTCT) of HIV services to health centers and hospitals [41]. Furthermore, the discrepancy might be due to sociodemographic and cultural differences.

The finding of this study revealed that residence of women is found to be determinants of time to first ANC booking. As the acceleration factor showed that pregnant women from rural residence have delayed time to first ANC booking than women’s from urban residence. This finding is in row with the study done in Uganda [40] and Nigeria [42]. This consistency points to the fact that women from urban residences might have better access to health care facilities than in rural areas. Women from urban areas may have had access to education, media exposure, and distance from health facilities did not pose a substantial barrier to early booking of ANC. Furthermore, pregnant women from the rural area might have poor maternal health care utilization due to lack of accessibility and availability of the service. It is pretty concerns that, maternal health care services are used differently in urban and rural areas. This disparity in time of ANC booking is common in many resource-limited contexts because of the unequal distribution of health services, generally favoring urban women. In addition to encouraging early booking, health facilities in rural, distant, and hard-to-reach communities should be refurbished with infrastructure, health care workers, and comprehensive maternal health care services as typically offered in urban areas.

The other important determinants of time to first ANC booking is educational level. Pregnant women who attend primary, secondary and higher education books first ANC earlier than women who had no education. This finding is in line with the study done Ghana [43], Nepal [44], Bangladesh [45] and Pakistan [46]. The possible explanation might be that the higher the educational level the better knowledge regarding the importance of timely initiation of ANC booking. This finding indicates a positive interaction between a mother's educational level and early ANC booking. Thus, policymakers should emphasize improving women's educational attainment by exposing them to educational materials and other awareness-creation campaigns, such as watching television and listening to the radio regarding maternal and child health information in parallel with other behavioral change communication media. Undoubtedly, exposure to health information will improve mothers' knowledge regarding the importance of timely initiation of ANC. This study also found that the wealth index has a significant effect on the time to first ANC booking. Women from wealth index categories poor had a prolonged time to first ANC booking than women from middle and rich wealth index categories. This finding is consistent with other previous study done in Ethiopia [47, 48] and Pakistan [46]. Women’s from wealthier household might have better autonomy, better educational level, and confidence in using maternal health care utilization [49–51]. Because of family related workload or other obligations, poorer women are less likely to obtain permission from their husbands and family members to visit a health facility for an ANC booking. Furthermore, concealed expenditures such as transportation fees contribute as impediments to timely booking of ANC by women from poor households. Aside from encouraging the early initiation of ANC, improving women's wealth level should also be a top priority. Since economic barriers have significant impact on maternal healthcare coverage, the concept of universal health coverage is fundamental. The disparity in maternal health care utilization related to wealth status is widespread in developing countries, especially in regions with low health insurance coverage. In Ethiopia, health insurance coverage is only 28% [52]. Thus, strategies to increase health insurance coverage are one of the methods to increase universal health coverage and thereby improve maternal health care utilization, including early initiation of ANC without any financial hardship.

The study's strength is that it employs national representative data, making it generalizable to all Ethiopian pregnant women. Because the data was self-reported, it might have been influenced by recall bias. Because the data sources are secondary, other potential predictors of time to first ANC booking are not quantified. Another issue is lack of a trend analysis.

## Conclusion

In this study, the median time to first ANC booking was four months. About 50% of pregnant women book their first ANC before 4 months. The time to first ANC booking in Ethiopia is higher than the WHO recommendation that states the first visit should be within 12 weeks [53]. The timing of the first ANC booking in Ethiopia was mainly influenced by the residence of women, mother educational level, and the wealth index.

Interventions might include improving women's educational attainment by exposing them to educational materials and other awareness-creation campaigns through communication media toward the consequence and importance of timely booking of ANC, particularly in rural settings. Increasing coverage of health insurance might also play a role in improving maternal health service utilization, including early initiation of ANC without any financial constraint. In addition, policymakers should focus on the establishment of income-generating activities and poverty reduction so as to minimize financial barriers for women from the poor wealth index, thereby improving the utilization of maternal services.

## Data Availability

The datasets generated and/or analyzed during the current study is publicly available and it was obtained from the 2019 Ethiopian Demographic and Health Survey. It is available through (https://www.dhsprogram.com)

## Abbreviations

AIC: Akaike information criteria
ANC: Antenatal care
BIC: Bayesian information criteria
EDHS: Ethiopia Demographic and Health Survey
EPMM: Ending Preventable Maternal Mortality
FMoH: Federal Ministry of Health
MMR: Maternal mortality ratio
NRERC: National Research Ethics Review Committee
PMTCT: Prevention of mother-to-child transmission
SSA: Sub-Saharan Africa
VIF: Variance inflation factor
